# Unraveling the Repertoire in Wiskott–Aldrich Syndrome

**DOI:** 10.3389/fimmu.2014.00539

**Published:** 2014-10-27

**Authors:** Sven H. Petersen, Anton Sendel, Mirjam van der Burg, Lisa S. Westerberg

**Affiliations:** ^1^Department of Microbiology Tumor and Cell Biology, Karolinska Institutet, Stockholm, Sweden; ^2^Department of Immunology, Erasmus MC, University Medical Center Rotterdam, Rotterdam, Netherlands

**Keywords:** Wiskott–Aldrich syndrome, WASp, B cell receptor, T cell receptor, immune repertoire diversity, next generation sequencing, spectratyping

Human immunology is entering the next frontier. With the fast developing technology, we can today sequence the whole genome from an individual in a relatively short time. It is now possible to decipher pathological mechanisms in immunological diseases, including primary immunodeficiencies, with high specificity. A particularly interesting aspect to study is the development and maintenance of the immune repertoire diversity and its consequences for disease progression. Until recently, a major difficulty in analysis of peripheral blood cells has been to sequence the locus encoding the T cell receptor (TCR) and the B cell receptor (BCR) in each cell. These receptors are assembled from a large array of V, (D), and J gene segments in a process that inserts and deletes nucleotides in the V(D)J junctions (Figures 1A,B). The antigenic specificity of the BCR and TCR is determined by the complementarity-determining regions (CDR) 1–3, where the CDR3 covers the junctions between the V, (D), and J segments and is the most variable part of the receptor (Figures 1A,B). To add to the complexity, B cells undergo further gene diversification in the peripheral germinal centers by class switch recombination and induction of somatic hypermutations.

**Figure 1:**
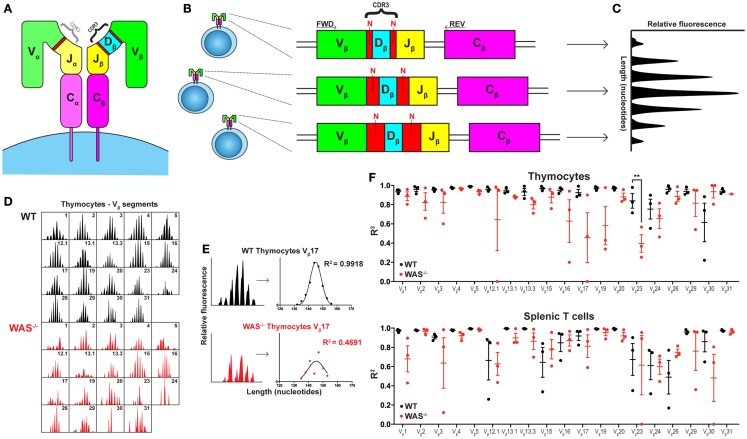
**Spectratyping analysis of TCRVβ repertoire in WT and WAS^−/−^ mice**. **(A)** Schematic of the TCR structure. The TCR is composed of an α and a β chain and the CDR3 regions determine most of the antigenic specificity. **(B)** V(D)J recombination results in varying CDR3 lengths. The size of the CDR3 region, comprising the segment junctions with a variable number of non-templated (N) nucleotides, differs between cells. **(C)** Spectratyping analysis. PCR products using TCRVβ specific primers (FWD) together with a Cβ primer (REV) were separated with capillary electrophoresis and analyzed for CDR3 transcript lengths using Peak Scanner™ Software v2.0. Each peak represents all cells expressing the same CDR3 length. In the healthy naïve T cell population, the CDR3 size distribution is Gaussian. **(D)** The thymocyte TCRVβ repertoires in one WT and one WAS^−/−^ mouse at 7 months of age. **(E)** Quantification of CDR3 size distribution. Non-linear regression analysis was performed on spectratyping data. An *R*^2^ value of 1 indicates a perfect Gaussian distribution. **(F)** TCRVβ–CDR3 size distribution in thymus and spleen of three WT and three WAS^−/−^ mice at 7 months of age. *R*^2^ values with mean ± SEM. **P = 0.0013.

Mutations in the Wiskott–Aldrich syndrome protein (WASp) cause the severe immunodeficiency disease Wiskott–Aldrich syndrome (WAS) (1, 2). WAS has been associated with numerous cellular defects and is termed a cell-trafficking disease of the immune system. WAS^−/−^ B cells are hyperactive and induce an autoreactive response, whereas WAS^−/−^ T cells are hyporesponsive and WAS^−/−^ T regulatory cells fail to suppress effector T cells (1, 2). Based on the important role of WASp for peripheral function, it has been somehow surprising that B and T cell development is intact as evident in normal progression through maturation stages in the bone marrow and thymus, respectively (2, 3). The role of WASp in creating a diverse BCR and TCR repertoire has until recently remained unknown. Since a skewed and oligoclonal BCR and TCR repertoire is linked to autoimmunity (2), a number of laboratories have now addressed the immune receptor repertoire in WAS patients. Two recent studies show that B cells from WAS patients have a decreased BCR repertoire, altered V gene usage, and decreased somatic hypermutation (4, 5). In T cells, Wada et al. showed already in 2005 that the TCRVβ repertoire was skewed in WAS patients older than 15 years, while younger WAS patients showed no repertoire skewing (6). This year, Braun et al. and Wu et al. showed that also young WAS patients often had a skewed TCRVβ repertoire (7, 8).

O’Connell and colleagues (9) have in the present investigation examined the BCR and TCR repertoire in WAS patients using next generation sequencing [NGS, see recent reviews in Ref. (10, 11)]. Using this technique, the authors collected a vast amount of data that allowed them to analyze the diversity of the receptor repertoire, including V(D)J segment usage, CDR3 size distribution, clonal expansions, and for BCRs; class switch recombination and frequency of somatic hypermutations. In B cells from WAS patients, the repertoire diversity tended to be lower than in controls, and the usage of some V heavy chain (VH) gene segments was skewed. However, WAS B cells had normal rate of somatic hypermutation. In T cells of WAS patients, clonal expansions were present in the memory CD4^+^ T cells and both in naïve and memory CD8^+^ T cells. The usage of TCRVβ gene segments tended to be skewed to a higher extent in WAS patient CD8^+^ T cells than in WAS patient CD4^+^ T cells. These results provide valuable information regarding the development and maintenance of the immune repertoire in WAS and importantly, describes alterations mainly in CD8^+^ T cells.

Interestingly, one out of the three patients in the study by O’Connell et al. showed increased VH4–34 expression that recognizes self-antigens (12). Two other recent studies have examined the BCR diversification in WAS patient B cells and also found overrepresentation of the VH4–34 gene (4, 5). This suggests that autoreactive B cells are expanded in WAS patients, and the current study indicates that these B cells may be present even before clinical signs of autoimmune disease. Simon et al. and Castiello et al. describe decreased somatic hypermutation in WAS patients while the three patients in the O’Connell study showed normal somatic hypermutation. This may reflect differences in clinical status of the patients both in regards to infections and presence of autoimmune disease. In the T cell compartment, Wada et al. used the classical technique of CDR3-spectratyping (Figure 1C) and showed a skewed TCRVβ repertoire in WAS patients over 15 years of age, while younger patients showed a normal TCRVβ repertoire. These results suggested that thymic diversification may be intact. The authors reasoned that the oligoclonal TCR repertoire could reflect accumulation of T cell clones specific to autoantigens or result from chronic infections (6). However, using the same technique, Braun et al. recently showed that even young WAS patients often had a skewed TCRVβ repertoire (7). The work by O’Connell et al. extends these studies by usage of NGS on sorted T cell subsets and identifies restriction in the TCRVβ repertoire mainly in CD8^+^ T cells from young WAS patients. A recently published study supports this finding and shows that WAS mutations in young patients differentially influence the TCR diversity of different T cell subsets as determined by both CDR3-spectratyping and NGS. Compared with age-matched healthy control subjects, TCR diversity of WAS patient cells was severely skewed in memory/effector CD4^+^CD45RO^+^ T cells and CD8^+^CD45RA^+^CCR7^−^ T_EMRA_ cells (13).

A limitation of the CDR3-spectratyping (Figure 1C) is that unique sequences are not identified. As a result, it is not possible to distinguish if a peak in the spectrum represents many unique sequences of identical length or one clonally expanded TCR. The highly detailed data output obtained from NGS enables intricate analyses regarding the relationship between unique sequences and total sequences. O’Connell et al. show that WAS patients have reduced diversity of unique BCR and TCR sequences, indicating decreased capacity to diversify the receptor repertoire in the bone marrow and thymus, respectively. As the authors discuss, reduced diversity of unique sequences may also reflect decreased survival of B and T cells in the peripheral organs. Another explanation may be that NGS do not yet provide full sequence coverage and that underrepresented BCR and TCRs are excluded.

In all studies of WAS patients, it is difficult to fully define the cause for disturbed BCR and TCR repertoires. The repertoire may show alterations due to aberrant development, exposure to chronic infections (that may vary with age and in different countries), and/or autoimmune disease commonly seen in older WAS patients. In patients, addressing these interesting aspects would require analysis of newborns with a family history of disease. In an attempt to investigate age-related alterations in thymic output and to exclude potential burden of infections, we examined the TCRVβ repertoire in thymus and spleen of 4 weeks young and 7 months old WAS^−/−^ mice housed in specific pathogen-free condition. In young WAS^−/−^ mice, the TCRVβ repertoire was indistinguishable to littermates controls and showed a Gaussian distribution reflecting the diverse TCR repertoire (unpublished data). In old mice, the TCRVβ repertoire of WAS^−/−^ mice showed more aberrations from the Gaussian distribution as compared to littermate control mice, both in thymus and spleen (Figures 1D–F). Our data suggest that in the absence of infections, autoantigens is the likely trigger of deviations from the diverse TCR repertoire in old mice. Moreover, the reduced TCRVβ repertoire diversity in thymocytes of old WAS^−/−^ mice support the notion raised by O’Connell et al. and Park et al. that thymic output is impaired in WAS (14).

Using NGS, valuable insights can be made in the study of the immune repertoire in human diseases such as WAS. The work by O’Connell and colleagues raises a number of intriguing questions, most importantly, how do relatively small changes in the BCR and TCR repertoire lead to severe disturbances in functionality of B and T cells? WAS^−/−^ B cells are intrinsically hyperactive and have increased homeostatic expansion in the periphery (15). This together with an altered BCR repertoire as shown in the present study and in previous studies (4, 5), suggests that B cells drive autoimmune disease in WAS. O’Connell et al. show that memory CD4^+^ T cells have a limited TCR repertoire and it has previously been shown that WAS^−/−^ CD4^+^ T cells can drive autoimmune colitis (16). The present study raises the intriguing possibility that also CD8^+^ T cells may be autoreactive with a limited TCR repertoire as is often seen in diseases with disturbed thymopoiesis such as Omenn syndrome (17). How this is related to previous findings of decreased cytotoxic capacity and reduced IFNγ production by WAS CD8^+^ T cells remains to be determined (18). Decreased production of IFNγ is characteristic of T_EMRA_ cells, a subset of CD8^+^ T cells that are terminally differentiated and functionally exhausted (19). T_EMRA_ cells are expanded upon chronic viral infection, a common clinical complication in WAS patients. It is possible that the restricted TCR repertoire of CD8^+^ T cells in WAS patients to a large extent is represented by an expansion of T_EMRA_ cells as shown in the present study by O’Connell et al. and in the recent paper by Wu et al. (9, 13).

What are the future challenges for NGS? An important question is to determine if peripheral blood lymphocytes represent B and T cells in tissues such as the spleen, lymph node, skin, and the gut. Peripheral blood B cells may represent as little as 2% of the body’s total B cells (11). A recent study raises the intriguing possibility that B cell development occurs at sites in the intestinal mucosa and strikingly, that the gut BCR repertoire is vastly different from the repertoire in the bone marrow (20). This suggests that the commensal microbes influence antibody diversification in the gut and that this may be an important aspect to consider in analysis of peripheral blood from patients. NGS is superior to all previous approaches in the coverage of sequences. Nevertheless, the number of unique BCR and TCR sequences by far exceeds the number of sequences obtained by NGS (normally 10^4^–10^5^ sequences as in the O’Connell study). Thus, with the current technology it may still prove difficult to obtain full coverage of the BCR and TCR repertoire. Another limitation is the reproducibility when analyzing rare sequences using alternative sequencing platforms. In one study, blood samples from the same donor were analyzed in parallel on the 454, Illumina, and Ion Torrent sequencing platforms (21). Profound discrepancies were revealed, with the three datasets differing significantly in terms of the diversity and relative abundance of clones within the overall repertoire.

This study identifies TCRs and BCRs that are clonally expanded in WAS patients. The next challenge is to identify the antigen recognized by a particular receptor. For B cells, Wardemann et al. described already in 2003 the method of single cell cloning and antibody expression to demonstrate that a large proportion of newly generated B cells in fact express self- and poly-reactive BCRs (22). This method has been particularly useful in identification of self-antigens in autoimmune disease. Antigen recognition by TCRs is more complex since TCRs can only recognize antigenic peptides presented by MHC molecules by numerous unpredictable contacts (10). To identify the antigen recognition by a cloned TCR, the TCR can be screened over a library of MHC-peptides in insect cells or yeast cells. By comparing the identified antigenic peptide to candidate antigens in disease, the pathological peptide can be identified successfully (23).

In conclusion, the present study begins to unravel the BCR and TCR repertoire in WAS patients and paves the road for a better understanding of the differences in response to treatment such as bone marrow transplantation and gene therapy, the latter currently in clinical trial (5, 7, 24).

## Conflict of Interest Statement

The authors declare that the research was conducted in the absence of any commercial or financial relationships that could be construed as a potential conflict of interest.

## Abbreviations

C, constant; CDR, complementary determining region; D, diversity; J, joining; N, non-templated; V, variable; WAS, Wiskott–Aldrich syndrome; WT, wildtype.
